# Supplementary material for: Are carbon emissions trading and green financial instruments synergistic? -Comprehensive quantitative research based on content analysis
Source: PLoS One. 2024 Mar 7;19(3):e0298601. doi: 10.1371/journal.pone.0298601 (PMC10919601; doi:10.1371/journal.pone.0298601)
Supplement: S1 Data — (ZIP) [file pone.0298601.s001.zip › Data/地级市Z_F工作报告.zip!/╕ú╜¿/╕ú╓▌╩╨/└┤╫╘╣1⁄2╓┌║┼í╛┬φ┐╦╩2╛▌═°í┐_2018.docx]

各位代表：

　　现在，我代表福州市人民政府，向大会报告政府工作，请予审议，并请市政协各位委员提出意见。

一、2017年工作回顾

　　2017年，在省委、省政府和市委的正确领导下，市政府全面贯彻落实党的十八届五中、六中全会和十九大精神，深入学习领会习近平新时代中国特色社会主义思想，聚焦“机制活、产业优、百姓富、生态美”，以“攻坚2017”“招商2017”等专项行动为抓手，迎难而上、攻坚突破、加压奋进，各项工作都取得了新的进展。预计全市地区生产总值突破7000亿元，增长8.5%；一般公共预算总收入突破1000亿元，增长7.7%；地方一般公共预算收入634亿元，同口径增长10.4%；固定资产投资5800亿元，增长12%；出口总额1510亿元，增长7.3%；实际利用外资123亿元，增长7%；社会消费品零售总额4181亿元，增长11%；居民消费价格总水平上涨1.1%；城镇居民人均可支配收入40859元，增长8%；农村居民人均可支配收入17817元，增长9%；城镇登记失业率2.43%。完成省下达的减排降碳任务。实现全国文明城市“三连冠”，荣获国家森林城市、中国领军智慧城市等称号。一年来的工作主要体现在：

　　（一）致力创新驱动，产业结构持续优化。出台推动新一轮经济创新发展十项政策49条措施，扶持实体经济发展壮大，新增上市企业11家，培育总部企业20家，6家企业进入中国民营企业500强，新增福建名牌产品49个、省级企业技术中心10家，完成技改投资870亿元、增长40.2%，引进高层次人才1322人。国家自主创新示范区福州片区建设扎实推进，新增国家级高新技术企业161家，新认定省级重点实验室4家、省级（企业）工程技术研究中心9家、省市众创空间61家，每万人有效发明专利拥有量14.3件、增长9.9%，顺利通过国家创新型城市评估。工业经济稳步增长，规上工业增加值2180亿元、增长8.1%，铭林钢构连江生产基地、罗源闽光60万吨高线升级改造等52个重大项目开工建设，京东方8.5代面板、经纬新纤等92个重大项目建成投产，福建奔驰、福耀玻璃等重点企业带动作用继续增强。新兴产业蓬勃发展，国家互联网骨干直联点正式开通，国家健康医疗、国土资源、旅游等行业大数据中心以及360、浪潮等136家关联企业落户中国东南大数据产业园，全国首家物联网开放实验室正式揭牌，全球规模最大的窄带物联网智慧水务商用项目进展顺利，中国物联网大会成功举办。国家服务业综合改革试点建设不断深化，服务业增加值超过3600亿元、增长11%，占GDP比重达50.5%，承办2017中国饲料工业展览会、第72届中国教育装备展示会等各类展会活动103场。农业基础继续夯实，农林牧渔业总产值910亿元、增长3.6%，新增全国休闲农业与乡村旅游示范县1个、市级农业产业化龙头企业60家、“三品一标”认证农产品53个，福州茉莉花茶品牌位居2017中国茶叶区域公用品牌价值第6位，完成生猪养殖场标准化改造163家，福清市畜禽养殖数字农业项目列入全国20个数字农业试点，全面完成罗源湾网箱养殖退养，粮食安全省长责任制考核位居全省第1位。

　　（二）致力提升品质，城乡面貌持续改善。成功进入国家15个城市总体规划编制试点行列，制定实施《福州中心城区空间发展规划》。城区水系综合治理全面展开，完成晋安河扩河、清障、清淤以及生态驳岸改造，新建井店湖、桂后溪湖、义井溪湖、洋下海绵公园、斗顶雨洪公园、八一雨洪公园，扩挖琴亭湖、温泉公园湖，整治城区易涝点45个，城市防涝能力大幅提升；埋设沿河截污管道70公里，清除内河淤泥211万立方米，清疏排水管网1020公里，打通断头河5条，城区已有43条内河基本消除黑臭；排查整治城区污染源2881个，取缔非法排污及无证经营企业102家，建设隔油池2597个，内河污染源得到有效控制；城区内河两侧各拆出6米空间，既为全线截污创造了条件，又新建滨河绿道20公里、串珠公园70个，水系周边环境得到改善；在全省率先成立城区水系联排联调中心，城区库、湖、池、河、闸、站基本实现一体化、智慧化管理；西湖整治12项措施、40个项目落实到位，整治污染源99个，清除湖体淤泥8.6万立方米，拆除沿湖违章建筑1.3万平方米，西湖的水质和周边环境明显改善；三江口、鹤林两个海绵城市试点片区建设有序推进；全市实现“河长制”全覆盖。完成连片旧屋区改造42个、566万平方米，整治提升老旧小区35个。地铁建设全面铺开，在1号线投入运营、2号线加快建设的同时，1号线延伸段、4号线、5号线、6号线、滨海快线等5条线路同步启动建设。组织实施第一批缓解城区交通拥堵软硬件项目252个，城区平均车速提高6.83%，日均堵情下降34%，交通拥堵排名从第19位下降到第32位。新改扩建市政道路203公里，修复沥青路面11.3万平方米，修补人行道35万平方米，增加公共停车泊位1.06万个，新建无害化公厕336座，拆除路面围挡24.5万平方米。完成火车南北站、城区13个出入口沿线环境整治和“一江一轴一线三中心”城区亮化提升工程。“全民动员、绿化福州”深入开展，造林绿化8.9万亩，种植大树21万棵，建成12条生态休闲步道、12个生态主题公园，鼓岭成为国家级旅游度假区,左海公园—金牛山城市森林步道荣获年度国内唯一的“国际建筑大奖”。城市空气质量位居全国74个重点城市第5位。滨海新城建设全面启动，长乐顺利撤市设区，新城综合医院、国际双语学校等136个启动项目按计划推进，新城核心区城市设计等30个专项规划顺利完成，临空经济区提升步伐加快。农村基础设施不断完善，新建改造农村公路190公里，建成乡镇污水处理设施19个、生活垃圾转运系统23个，江北城区山洪防治及生态补水工程、平潭及闽江口水资源配置工程等71个重大水利项目完成年度投资计划，建成安全生态水系6条。新建新农村“幸福家园工程”示范村（创建村）425个、美丽乡村111个。福清龙田镇入选第二批全国特色小镇，新增4个省级特色小镇。全市拆除“两违”918万平方米。

　　（三）致力改革开放，发展活力持续释放。福州新区完成重点项目投资1915亿元，占年度计划的112%，对全市经济增长贡献率超过25%。自贸试验区福州片区新增企业8277户，推出3批44项创新举措、其中全国首创12项，江阴口岸集装箱吞吐量增长25%、整车进口突破1万辆。推进“三去一降一补”，全面取缔“地条钢”企业，商品房库存同比下降27.2%，不良贷款率比年初下降0.19个百分点，减税降费73亿元，33个补短板工程包完成投资257亿元、占年度计划的128%。加快“放管服”改革，取消行政审批事项68项、下放22项，确定“最多跑一趟”和“一趟不用跑”事项1334项、占总量的93%，在全国率先实行行政审批全流程电子证照应用和不动产证“网上办理”，在全省率先开展“证照分离”改革和“减证便民”专项行动，荣获中国“互联网+政务服务”创新奖、全国行政服务大厅典型案例“综合十佳”。国企改革稳步推进，组建地铁集团、金控集团、电子信息集团，海峡环保公司成功上市。国家社会信用体系建设示范城市创建加快推进，信用平台和网站建设水平跃居全国第6位。国家生态文明试验区建设完成17项改革任务，重点生态区位商品林赎买、碳汇交易、林地占补平衡等试点持续开展。在全省率先实施城市管理综合执法体制改革。基本完成农村土地承包经营权确权登记工作，晋安区确权成果在全省率先通过农业部初检并入库。全市耕地流转总面积58万亩，完成闽侯县国家级农村集体资产股份权能改革试点。对外开放步伐加快，新批千万美元以上外（台）资项目140项、境外投资项目26项。成功举办金砖国家政党智库和民间组织论坛、世界城地组织2017年亚太区执行局和理事会会议、第九届世界华文传媒论坛、第十九届海交会、2017渔业周·渔博会、第三届海丝国际旅游节、第四届丝路国际电影节等重大会议和活动，发起成立“21世纪海上合作委员会”。成功举办第五届海峡青年节、海峡两岸合唱节等41项特色对台交流活动，海峡青年交流营地正式启用。与澳大利亚霍巴特市、菲律宾马尼拉市缔结友城关系。闽东北、闽浙赣皖、泛珠三角区域协作不断拓展。

　　（四）致力共享发展，民生福祉持续增进。全市各级财政用于民生支出746.64亿元，占一般公共预算支出的79.4%。完成20项75件为民办实事项目。新改扩建40所中小学、13所公办幼儿园，启动55个智慧校园建设，普惠性幼儿园学额覆盖率提高至74.87%，省级达标中职学校实现全覆盖，“安全教育实验区”建设、中小学德育工作走在全国前列。市图书馆新馆建成开放，完成70个乡镇（街道）综合文化站和499个村（社区）综合文化服务中心达标改造，上下杭、烟台山、冶山历史文化街区（风貌区）保护修复进入实质性实施阶段。组织编纂《福州通史》，成功举办第六届闽都文化论坛。孟超肝胆医院门诊综合楼、市中医院新病房综合大楼等卫生领域重点项目加快建设，分级诊疗制度初步构建，家庭医生签约服务全面开展，在全省率先实现医联体基层医疗机构全覆盖。全面两孩政策平稳实施。群众体育和竞技体育协调发展，参加全民健身运动人数超过100万，成功举办中华龙舟大赛、中国羽毛球公开赛、环福州·永泰国际公路自行车赛、福州国际马拉松赛等大型体育赛事。在全省率先实施全民参保登记计划，退休人员养老金待遇、城乡低保补助标准、特困人员供养标准、城乡居民医保人均筹资水平稳步提高。新建农村幸福院261所、社区居家养老服务照料中心37个，提升居家养老服务站24个。城镇新增就业13.5万人，转移农业富余劳动力3.8万人。房地产市场保持稳定，建成保障性安居工程1.88万套，新增公租房配租1万套，启动共有产权住房建设试点。完成市县两级两批722个重大安全隐患点整改，安全生产形势稳中向好。“平安福州”建设深入推进，全市刑事案件发案数同比下降20.2%，平安建设知晓率、执法工作满意率均位居全省第1位，群众安全感率达94.55%。闽清、永泰灾后重建任务全面完成。所有建档立卡扶贫对象可如期脱贫，永泰省级、闽清市级扶贫开发重点县可实现“摘帽”，成立扶贫发展基金会，建成福州市惠民资金网。援宁援藏援疆、与定西市扶贫协作等工作有效开展。民族宗教、信访、行政复议、司法、仲裁以及国防动员、双拥共建、民兵预备役建设、海防、人防、反走私、爱国卫生等工作继续加强。广播影视、新闻出版、哲学社会科学、统计、科普、气象、防震、地方志、档案、老龄、妇女儿童、残疾人、慈善等各项事业全面发展。

　　（五）致力攻坚突破，发展后劲持续增强。围绕经济社会发展的重点问题和群众关注的热点问题，扎实开展系列专项行动。“攻坚2017”行动硕果累累，破解阻碍项目落地的各类矛盾和问题4158个，交地14.51万亩，拆迁1341.55万平方米，动建项目448个，竣工项目238个，福厦客专、宝钢德盛二期等一批重大项目得以顺利落地，1.87万户逾期安置户得以顺利回迁，3.47万亩批而未供土地、1.44万亩供而未建土地得以顺利处置。“招商2017”行动成绩斐然，落地招商项目2647项、总投资6212亿元，其中，投资30—100亿元的项目35个，投资100亿元以上的项目2个，比特大陆人工智能、西门子研发中心等一大批世界级制造业项目相继签约、落地，吸引“回归工程”项目83项、总投资746亿元。“海上福州”建设行动亮点纷呈，落实172个支撑项目，完成投资406亿元，江阴港区11号、12号泊位提前投入运营，福州港新增4条国际集装箱班轮航线，福清核电4号机组、华能罗源火电厂等一批重大临港工业项目竣工投产，中国邮轮旅游发展实验区、国家海洋经济示范区落户福州。服务业跨越发展行动卓有成效，出台配套政策23项，动建项目55个，竣工项目34个，完成投资537亿元，新增省级服务型制造示范企业9家、省级服务型制造公共服务平台4个，入选国家供应链体系建设首批重点城市，成为全国第三个设立无现金联盟的城市，宜家家居、“八仙过海”旅游文化综合体等一批项目落地。全市接待游客6742.66万人次，增长22.1%。

　　（六）致力提升效能，作风建设持续加强。扎实推进“两学一做”学习教育常态化制度化，深入开展“学俊波、我践行”主题实践活动。认真践行“马上就办、真抓实干”精神，大力推行一线考察、一线巡察，充分发挥市级7个专项督查组和重大项目督办工作机制的督导作用，不等、不停、不看、不混日子、不当懒汉，干部干事创业精气神焕然一新，在全市范围内形成了力争上游、不甘落后、比学赶超的浓厚氛围。召开50多场重点企业市领导专题协调会，为企业解决生产经营中遇到的具体问题834个。全面贯彻中央八项规定精神，驰而不息整治“四风”，长乐移风易俗经验在全省推广。行政监察、审计监督力度加大，反腐倡廉深入推进。认真执行市人大及其常委会决定决议，自觉接受市人大及其常委会的法律监督、工作监督。自觉接受市政协的民主监督，积极支持市政协开展民主协商，创新市政府与市政协专题协商方式。提请市人大常委会审议地方性法规草案4件，制定政府规章和规范性文件51件。办复404件省、市人大代表建议和589件省、市政协提案，满意率分别达97.52%、100%。

　　各位代表，成绩来之不易，这是省委、省政府和市委坚强领导的结果，这是全市人民团结奋斗、社会各界大力支持的结果。在此，我代表市人民政府，向全市人民，向人大代表、政协委员、各民主党派、工商联、无党派人士、各人民团体、离退休老同志和社会各界人士，向中直、省直机关企事业单位和驻榕部队指战员、武警官兵、公安政法干警，向关心支持福州发展的港澳台同胞、海外侨胞和国际友人，表示衷心的感谢！

　　在总结成绩的同时，我们也清醒地看到当前福州发展中还存在不少困难和问题，主要是：发展不平衡不充分、发展活力动力不足、发展质量效益不高的问题仍然比较突出；工业特别是制造业相对薄弱，龙头企业不多不强，新兴产业有待培育，新的增长点尚未形成；生态环境保护任务依然繁重，城区水系综合治理需要进一步强化；民生领域存在较多短板，交通、教育、医疗、居住、养老等方面与人民群众的需求还有较大差距。此外，一些领域腐败问题时有发生，还存在少数干部懒政怠政、推诿扯皮等现象。对此，我们要采取有效措施，认真加以解决。

二、2018年工作安排

　　各位代表，根据市委统一部署，今年市政府工作总体要求是：全面贯彻党的十九大精神，以习近平新时代中国特色社会主义思想为指导，坚持稳中求进工作总基调，坚持新发展理念，以供给侧结构性改革为主线，围绕推动质量变革、效率变革、动力变革，把高质量发展和实现赶超有机统一起来，把握“五区叠加”战略机遇，马上就办、真抓实干，持续提振干事创业精气神，统筹推进稳增长、促改革、调结构、惠民生、优生态、防风险各项工作，坚决打好三大攻坚战，加快建设环境更美、品质更好、功能更全、服务更优的有福之州。

　　今年全市经济社会发展主要预期目标是：地区生产总值增长9.4%左右，地方一般公共预算收入同口径增长8%，固定资产投资增长12%，社会消费品零售总额增长11.5%，出口总额、实际利用外资增幅高于全省平均水平，居民消费价格总水平涨幅控制在3%左右，城镇居民人均可支配收入增长8%，农村居民人均可支配收入增长9%，城镇登记失业率控制在3.5%以内，确保完成省下达的节能减排降碳任务。

　　为实现上述预期目标，重点抓好以下五个方面工作：

　　（一）以“机制活”为引擎，深化改革开放，切实增强发展新动能。

　　加快福州新区全域开发。完善新区行政管理体制机制，落实新区发展规划。加快三江口、闽江口、福清湾、江阴湾等重点区域和组团的开发进度。推动马尾大桥、东南快速路等联接通道建设进程。促进琅岐岛综合文旅等重大项目尽快落地，打造国际生态旅游岛。共建福清飞地产业园，推动福州新区与平潭两地联动发展。依托江阴化工新材料基地、中铝东南沿海铝精深加工基地等载体，打造具有核心竞争力的产业体系和产业集群。推动学校、医院等优质资源和公共服务、商业设施向新区布局。新区重点项目年度计划投资2000亿元以上。

　　推进自贸试验区先行先试。落实省、市新增试验任务，推出30项以上创新举措。推动跨境电商基地、先进制造业技术服务中心等重点平台建设，促进银河汽车产业园、北美西洋参进口分拨中心等项目尽快投入运营。发布第四批金融创新案例，建设两岸金融创新合作示范区。提升福州保税港区和福州出口加工区建设水平。争取获批福州空港综合保税区。申报自由贸易港，营造国际化营商环境。争取开通“榕欧”铁路班列、空中航线，构建海陆空“三位一体”的跨境快捷物流体系。

　　深化重点领域和关键环节改革。推进供给侧结构性改革，坚决淘汰落后产能和工艺，鼓励企业增品种、提品质、创品牌，降低实体经济成本。完善行政管理体制，实现政府部门权责事项动态调整和精细化管理。推行综合执法改革，强化“双随机一公开”跨部门联合监管，逐步实现“多帽合一”。完成环保机构垂直管理改革。出台国企改革方案，理顺国资国企管理体制机制，实现国有资产保值增值。支持民营企业发展，落实促进民间投资政策，严格市场准入负面清单制度，破除歧视性限制和各种隐性障碍，引导社会资本进入基础设施、社会事业等领域。开展中小微企业综合金融服务试点，推动海峡银行、福州农商银行设立普惠金融部门，强化防控措施，守住不发生区域性系统性金融风险的底线。

　　构建全面开放新格局。坚持全员招商、小分队招商、“一把手”招商等工作机制，实施“回归工程”和“走出去”战略，办好第二十届海交会等招商活动，千方百计引进一批对经济发展有重大拉动作用、能够产生深远影响的大项目、好项目。办好第四届海丝国际旅游节等活动，推进“海上丝绸之路：福州史迹”申报世界文化遗产。办好第六届海峡青年节，复建福州台湾会馆，鼓励和支持台湾青年来榕创业就业，扶持培育马尾至马祖、黄岐至马祖两条海上客运航线，建成马尾对台客运码头。做好新华侨华人和华裔新生代工作。强化与港澳在金融、物流、文化创意等领域的合作。深化国际友城合作。加强泛珠三角、闽浙赣皖、闽东北区域协作，抓好援宁援藏援疆工作，继续做好与定西的扶贫协作以及与渭南、延安、吉安的对口合作。

　　优化投资发展软环境。完善新一轮经济创新发展十项政策，落实各类惠企措施。深化简政放权，抓好全国“互联网+政务服务”综合试点工作，健全“多规合一”“多评一表”“多图一审”、套餐式服务等创新机制，强化重大项目储备、预审、报批、建设、管理全流程代办服务，扩大政务数据共享应用，让信息数据“多跑路”，让办事群众“少跑腿”。坚持并完善重点企业市领导专题协调会制度，“一企一议”为企业排忧解难。

　　（二）以“产业优”为导向，推动高质量发展，着力构建现代化经济体系。

　　发展特色现代农业。坚持质量强农、绿色兴农，大力发展设施农业、智慧农业、休闲农业、生态农业，打造“一区三圈三带七产业两基地”都市型现代农业格局。落实粮食安全省长责任制，确保粮食种植面积稳定在150万亩、总产量达52万吨。强化县乡土地流转平台建设，推进土地承包经营权流转。培育壮大农产品加工、流通等农业产业化龙头企业。提升茉莉花茶、福州橄榄、福州金鱼等品牌效应。推进台湾农民创业园等现代农业园区建设。发展森林康养、林下经济、花卉苗木等绿色富民产业。

　　壮大先进制造业。实施“中国制造2025”福州行动计划，促进实体经济和数字经济、工业化和信息化、制造业和现代服务业深度融合，推动制造业向中高端迈进。改造提升纺织化纤、冶金建材、轻工食品等传统产业，形成1个三千亿元以上、2个两千亿元以上、2个一千亿元以上的产业集群。引导企业加大技改投入，实施100项以上重点技改项目，完成技改投资900亿元以上。加快项目建设进度，推动京东方柔性面板、高意科技半导体、康乃尔MDI等项目尽快开工，促进唐源24万吨锦纶、瑞玻玻璃等项目尽快竣工，加快申远新材料聚酰胺、法液空液化煤气化、旭福光电玻璃基板等项目尽快达产。落实品牌发展战略，支持企业收购国际知名品牌，培育一批名牌产品。加大扶持企业上市力度，力争有10家以上企业进入上市程序。落实工业园区单列统计、单列考核等体制机制改革事项，完善基础配套设施，推进园区规模化、差异化、专业化发展。开展质量提升行动，推进全国质量强市示范城市建设。

　　提升现代服务业。用足用好鼓励新引进企业总部的四条措施，力争新引进企业总部或职能机构80家以上。优化提升东街口、万宝等传统商圈，培育壮大东二环泰禾广场、红星爱琴海等新兴商圈，建设海西现代金融中心区、利嘉国际商业城等服务业集聚区。推进国家级网络市场监管与服务示范区建设，规范电商产业发展。建设福清公路港等一批物流配送中心、冷链物流基地，开展无车承运人试点工作。支持阿里巴巴“盒马鲜生”、永辉“超级物种”、泰禾“大有码头”等新零售业态发展，打造全国知名的新零售之都。继续推进制造业主辅分离，力争新增制造业主辅分离企业10家以上。鼓励发展绿色金融、普惠金融，构建“基金小镇”等特色金融业集聚区。打响闽都文化、温泉养生、滨江滨海、清新生态等旅游品牌，完成鼓岭旅游度假区提升改造工程，加快长乐下沙、罗源湾、环福清湾等滨海旅游度假区建设进度。办好第83届全国汽车配件交易会等大型展会。

　　培育战略性新兴产业。实施高成长性企业、高新技术企业培育工程，发展一批具有创新能力的排头兵企业。发挥中国东南大数据产业园、中国·福州物联网产业基地等专业园区的聚集效应，推进贝瑞基因、软通动力、物联网产业孵化中心等项目建设，大力发展大数据、物联网产业。加快三峡海上风电装备产业园、永泰抽水蓄能电站以及福清核电5号、6号机组等项目建设，大力发展新能源产业。加快坤彩珠光材料、旭成复合隔膜、金强建筑新材料等项目建设，大力发展新材料产业。加快克里贝尔生物医药、博奥基因检测中心、宏东海洋生物产业园等项目建设，大力发展生物医药产业。打造青口投资区、闽台（福州）蓝色经济产业园等新能源汽车产业基地，推动北汽福建奔驰新能源汽车、东南汽车（三期）、雪人股份氢能源等项目尽快落地，新建充电桩3400个，推广应用物流专用电动汽车2500辆，大力发展新能源汽车产业。

　　发展海洋经济。落实“海上福州”发展战略，启动国家级海洋经济示范区建设。重点推进江阴港区6—9号泊位、罗源湾可门作业区1—3号和14号泊位等项目建设，壮大港口经济。建立海洋经济重点项目库，实施首批16个“十三五”海洋经济创新发展示范项目。推动近海网箱养殖向陆地工厂养殖及深水抗风浪大网箱养殖转化，提升鉴江海洋生物产业园等特色园区，培育海洋特色产业。完善加快发展远洋渔业十条措施，建造2艘南极磷虾捕捞加工船，推进深海时代产业园等渔业园区建设，做大远洋渔业。探索海域资源市场配置与产权交易创新机制，构建海洋产权交易平台。办好渔业周·渔博会。推进福清黄官岛、长乐东洛岛等无居民海岛合理开发和福州海洋研究院建设，加强海洋保护和管理。

　　加快创新驱动。推进国家自主创新示范区福州片区建设，促进院所、高校、企业、创客等各类创新主体协作融通。落实科技成果转移转化若干措施和创新型产业用地实施意见，促进科技资源和创新要素向企业集聚。发挥企业创新主体作用，力争新增国家级高新技术企业100家、省级众创空间10家、市级众创空间20家。继续创建中国软件名城、国家信息消费示范城市、国家数字家庭应用示范产业基地，推进软件和信息技术服务业加快发展。抓好国家双创示范基地、海西创业基地、清华—福州数据技术研究院等平台建设，吸引国内外一流大学、科研机构及央企、跨国公司的研发机构落户福州。开展新一轮国家知识产权示范城市建设，力争每万人有效发明专利拥有量15件以上，新增各级知识产权示范优势企业40家。深入推进闽都人才集聚工程，实施“五个一千”人才行动计划，建设国际人才交流中心等一批人力资源服务集聚区。

　　（三）以“百姓富”为根本，补齐民生短板，不断提高群众的获得感、幸福感和安全感。

　　优先发展教育事业。新改扩建中小学34所、公办幼儿园13所，建设智慧教室500间，改造校舍25.2万平方米、运动场2.92万平方米。进一步规范办园行为，力争学前三年入园率达98%以上，普惠性幼儿园学额覆盖率达77%以上。实施消除义务教育阶段大班额专项规划，探索建立随迁子女积分入学管理办法，调整高中招生和民办初中招生办法。优化普通高中布局，推进普通高中学生综合素质评价改革，提升全市总体成绩。加强师德师风建设，完善教育师资补充和高素质教育人才培养机制。加快发展现代职业教育，推进“校中厂”“厂中校”建设，创建高水平公共实训基地，培养紧缺急需的技术技能型人才。支持在榕高校发展，支持市属高校内涵式发展，支持民办教育发展，办好特殊教育、网络教育、老年教育、继续教育、终身教育。加快与天津大学联合创办国际合作高等院校。

　　推动文化繁荣。规划建设市群艺馆新馆、第二工人文化宫、少儿图书馆新馆，保护修复上下杭、烟台山、冶山等历史文化街区（风貌区），动建闽台（福州）文化产业园、闽越水镇等项目，启用海峡文化艺术中心，优化三坊七巷街区业态，办好海丝情—中国梦、新福州人歌手大赛等群众文化活动。打造AR、VR等新兴文创产业，建设海西动漫创意之都。挖掘优秀传统特色文化，扩大闽都文化影响力。促进哲学社会科学、新闻出版、广播影视、文学艺术等事业繁荣发展。

　　建设健康福州。实施医疗卫生项目“333”工程，支持省儿童医院、妇产医院、疾控中心等省级项目建设，加快市一医院新外科病房大楼等13个市级项目建设，新建18所基层中医馆、12个市级医疗质量控制中心。深化公立医院综合改革，实现“药、价、保”联动，努力解决“看病难看病贵”问题。健全分级诊疗体系，完善家庭医生签约服务保障机制，推进紧密型医共体、医联体建设。提升重大疾病和突发急性传染病应急处置能力。持续开展爱国卫生运动，做好国家卫生城市复查迎检。落实计划生育目标管理责任制，强化妇幼健康服务保障。继续办好中华龙舟大赛、环福州·永泰国际公路自行车赛、福州国际马拉松赛等大型赛事，全力备战第十六届省运会。完善全民健身设施，推动各类场馆免费或低收费开放。

　　完善社会保障体系。推进参加社会保险法定人员全覆盖，提高退休人员养老金待遇、城乡低保补助标准、城乡居民医保人均筹资水平。推动医养结合，新建社区居家养老服务照料中心21个、农村幸福院200所，建成投用市本级、福清、晋安和闽侯等社会福利中心，推进福建海峡健康养老中心、静馨嘉园养老中心等项目建设。发展社会救助、慈善和残疾人事业。加快建立多主体供应、多渠道保障、租购并举的住房保障制度，新开工及新增供应租赁住房和共有产权住房1万套。抓好“菜篮子”工程，保持物价稳定。推进国家食品安全示范城市建设，让人民吃得安全放心。促进高校毕业生、农民工、就业困难人员和退役军人等重点群体就业创业，实施职业技能培训4万人次，城镇新增就业12.2万人，转移农业富余劳动力3.8万人，城镇失业人员再就业0.85万人。完善政府、工会、企业共同参与的协商协调机制，构建和谐劳动关系。

　　巩固脱贫攻坚成果。建立稳定脱贫的长效机制，探索产业帮扶、就业帮扶、智力帮扶、金融帮扶、健康帮扶等新模式，坚持贫困线和低保线“两线合一”，确保“三保障”各项政策落实到位。启动第六轮驻村帮扶工作，实施82个薄弱村村级集体经济发展项目。加强革命老区、少数民族贫困村、经济薄弱村以及100人以上民族自然村的基础设施建设。继续开展榕商公益光彩行动，用好市扶贫发展基金，调动社会各方力量参与扶贫事业。

　　维护社会安定稳定。深化“平安福州”建设，完善社会治安防控体系，实施“雪亮工程”，继续推进高清视频监控探头建设。健全重大决策社会稳定风险评估机制和突发事件应急处置机制，依法处理信访事项，完善人民调解、行政调解、司法调解等多元化联动体系。推广鼓楼军门社区和长乐百户村的经验做法，推动社会治理重心向基层下移。完成村（居）换届选举。开展“七五”普法。做好社区矫正工作。实施重点领域安全专项整治，坚决防范和遏制重特大安全事故发生。加强民族工作，依法管理宗教事务。巩固提升双拥模范城创建成果，全力支持驻榕部队改革调整和各项建设，加强国防教育、国防动员和海防、人防、反走私等工作，推进军民融合深度发展。支持工会、共青团、妇联、残联等人民团体发挥更大作用。做好防震减灾、统计、科普、气象、保密等工作。

　　提升城市文明水平。培育和践行社会主义核心价值观，巩固文明城市创建成果，持续深化文明城市、文明村镇、文明单位、文明校园、文明家庭创建工作。推进“书香榕城”建设，办好福州读书月系列活动。扎实开展移风易俗、弘扬时代新风行动，大力倡导文明风尚。依托“讲文明树新风”公益广告、“福州微文明”微信公众号等宣传平台，加大文明教育力度。完善志愿服务工作制度和平台建设，提升志愿服务实效。推进诚信建设制度化，完善公共信用信息平台，打造“信用福州”。

　　（四）以“生态美”为目标，践行绿色发展，精心打造美丽福州。

　　提升城市功能品质。编制新一版福州城市总体规划，实现市域“一张蓝图”。持续推进城区水系综合治理，统筹抓好内涝治理、水质提升、水系周边环境整治等各项工作，启动白马河治理，开挖晋安湖、旗山湖，加快江北城区山洪防治及生态补水工程建设进度，排查修复四城区排水管网1000公里，埋设沿河截污管道156公里，改造生态驳岸73公里，新建串珠公园98个，建成晋安河畔旅游观光带，增加滨河绿地109万平方米、滨河步道270公里，提标改造洋里、祥坂等13座污水处理厂，完成第二批国家海绵城市试点建设任务。全力推进51个片区、1041万平方米、3.82万户连片旧屋区改造。完成300个老旧小区综合整治。实施园林绿化“五个提升”工程，完成绿化福州项目49个，实现省级园林城市（县城）全覆盖。强化城市精细化管理，建设城市运营管理中心、共享单车管理平台、出租车管理系统，加强城市立面整治，规范户外广告设置，推进“厕所革命”，新建、改建无害化公厕377座，在城区和大学城开展第二阶段生活垃圾分类试点。持续治理“两违”、非法采砂等问题。

　　增强城市承载能力。加快绕城高速东南段、长平高速、长福高速、莆炎高速等4条高速公路和福厦客专建设步伐。提速地铁1号线延伸段、2号线、4号线、5号线、6号线建设。实施洪山桥和洪塘大桥拓宽改造、福泉连接线、道庆洲大桥等市政路桥工程，新改扩建市政道路160公里以上，完成齐安路、同德路等41条道路“白改黑”。建成福马路、万新路等地下综合管廊6公里以上。实施第二批385个缓解城区交通拥堵软硬件项目。新增公共停车泊位7500个，更新新能源公交车250辆，新建公交场站2个，改造公交停靠站100个。新改建供水管网200公里、燃气管道122公里，推进城区缆线下地。新改扩建红庙岭垃圾发电厂（三期）以及餐厨垃圾、危险废弃物无害化处理设施等7个项目。

　　加快滨海新城建设。提速滨海快线、东南快速通道等设施建设，完成机场第二轮扩容工程，启动机场第二跑道项目。开工建设“数字中国”会展中心、海峡青少年活动中心、东湖湿地公园、滨海森林景观防护带以及新城综合医院、高端酒店、人才公寓等45个配套设施。推进国家健康医疗大数据中心、国土资源大数据应用中心、交通大数据中心以及数字福建政务云、商务云、超算中心（二期）的产业应用。培育航空物流、航空维修、轨道交通装备等临空产业，促进临空经济区建设实现质的提升。发展总部经济、金融商务等现代服务业，打造国际滨海旅游胜地。

　　实施乡村振兴战略。按照产业兴旺、生态宜居、乡风文明、治理有效、生活富裕的总要求，制定乡村振兴战略规划，全面推进美丽乡村建设。新建300个美丽乡村，培育100个美丽乡村示范村。铺设乡镇污水管网91.2公里，完成688个行政村污水治理、52个行政村垃圾治理，新建改造水冲式公厕400座，整治“裸房”2000栋以上。实现县城组团、中心镇、乡村的客运网络全覆盖。加强古村落、古建筑的保护修复，继续推进历史文化名镇、名村、少数民族特色村寨建设。抓好福清市国家级新型城镇化综合改革试点，新建3个以上省市级特色小镇。推进农垦改革发展，基本完成土地使用权确权登记发证。稳步推进农村集体产权制度改革，开展经营性资产股份合作制试点。强化龙头企业、农民合作社、家庭农场等各类经营主体之间的联结机制，壮大农业产业化联合体。扶持发展“淘宝村”，加快培育农村电商。发挥福州市惠民资金网的作用，保障农民合法权益。

　　深化生态文明体制改革。加快生态文明试验区建设，形成一批可复制、可推广的制度创新成果。探索连江自然资源资产管理制度创新试点经验，推动自然资源资产的量化、登记和交易。建立以企业为主体的排污权交易制度，推进碳排放权交易、用能权交易。启动生态环境损害赔偿制度改革，实施重金属、危险废物等高风险行业环境污染责任保险试点。加大企业环境信用评价和联合奖惩，建立环境违法企业“黑名单”制度。加大对重点生态功能区的补偿。强化绿色发展绩效评价考核。落实领导干部自然资源资产离任审计。

　　打好污染防治攻坚战。划定生态保护红线，严禁引进高耗能、高污染、高排放项目。实行能源和水资源消耗、建设用地等总量和强度“双控行动”，强化县（市）区污染物减排刚性约束。加强工业污染源治理，实施钢铁、化工等高耗能行业节能循环经济项目30项。推广绿色建筑、装配式建筑，实现城镇绿色建筑占新建建筑50%以上，争创国家装配式建筑示范城市。加强施工扬尘、道路扬尘、机动车尾气等污染整治，力争空气质量在全国74个重点城市排名“保八争四”。加强重点流域水污染防治，实现县级以上重点水源水质自动监测全覆盖。加强湿地保护，推动红树林海岸公园建设。开展第二次全国污染源普查工作。

　　（五）以“马上就办”为准则，提振精气神，努力建设人民满意的政府。

　　坚定政治立场。认真贯彻落实党的十九大精神，牢固树立“四个意识”，不断增强“四个自信”，深入开展“不忘初心、牢记使命”主题教育，严守政治纪律和政治规矩，坚决维护党中央权威和集中统一领导，始终同以习近平同志为核心的党中央保持高度一致。坚决服从市委在推动福州发展中总揽全局、协调各方的领导作用，以实际行动确保党中央国务院、省委省政府和市委各项决策部署落到实处。

　　坚持依法行政。严格按照法定权限和程序用权履职，完善政府议事规则和决策程序，做到法无授权不可为、法定职责必须为。自觉接受市人大及其常委会的法律监督、工作监督和市政协的民主监督，创新监督和协商方式，提高办理人大代表建议、政协提案和协商意见的落实率、满意率。广泛听取各民主党派、工商联、无党派人士和各人民团体、社会各界人士的意见建议。深入推进政务公开，主动接受舆论监督和群众监督。

　　强化责任担当。坚决贯彻市委“担当负责、提速增效”的要求，落实一线考察、正向激励，为敢于担当、敢于碰硬、不谋私利的干部撑腰鼓劲，让广大干部想干事、能干事、干成事，全面提振干事创业精气神。大兴调查研究之风，坚持在一线了解实情，在一线发现问题，在一线解决问题。大力倡导说了就办、定了就干、干就干好的实干精神，不驰于空想、不骛于虚声，坚决整治“庸懒散拖”，不断提高政府执行力和战斗力。

　　创新工作方法。深入开展“抓项目促发展”专项行动，全体动员，集中力量，一切围绕项目，一切聚焦产业，通过大批产业项目的引进、大批产业项目的落地、大批产业项目的投产，推动全市经济实现跨越式发展。运用项目工作法，围绕项目的落实，构建严格的督查体系，明确时限，落实责任，奖优罚劣，让项目涉及的资金、用地、用林、用海等各类问题，都有人跟踪，有人协调，有人解决。

　　加强廉洁建设。坚持把纪律和规矩挺在前面，以制度管权、管事、管人，把权力关进制度的“笼子”。严格落实中央八项规定精神，坚持不懈反“四风”，扎扎实实改作风。切实履行党风廉政建设主体责任，无禁区、全覆盖、零容忍惩治腐败。强化审计监督，严明财经纪律。构建“亲”“清”新型政商关系，守住从政为民的“压舱石”，筑牢拒腐防变的“防火墙”。

　　各位代表，“幸福都是奋斗出来的”。新的征程任重道远，新的使命催人奋进。让我们高举习近平新时代中国特色社会主义思想伟大旗帜，更加紧密地团结在以习近平同志为核心的党中央周围，在省委、省政府和市委的坚强领导下，不忘初心、牢记使命，勇往直前、搏击奋进，为开创新时代有福之州建设新局面、谱写全市人民美好生活新篇章而努力奋斗！
